# Tissue-Specific Transcriptomics in the Field Cricket *Teleogryllus oceanicus*

**DOI:** 10.1534/g3.112.004341

**Published:** 2013-02-01

**Authors:** Nathan W. Bailey, Paris Veltsos, Yew-Foon Tan, A. Harvey Millar, Michael G. Ritchie, Leigh W. Simmons

**Affiliations:** * Centre for Biological Diversity, School of Biology, University of St Andrews, St Andrews, Fife KY16 9TH, United Kingdom; † Centre for Evolutionary Biology, School of Animal Biology (M092), The University of Western Australia, Crawley 6009, Australia; ‡ Centre for Comparative Analysis of Biomolecular Networks, The University of Western Australia, Crawley 6009, Australia

**Keywords:** field cricket, gryllid, reference transcriptome, *Teleogryllus oceanicus*, tissue-specific transcriptome

## Abstract

Field crickets (family Gryllidae) frequently are used in studies of behavioral genetics, sexual selection, and sexual conflict, but there have been no studies of transcriptomic differences among different tissue types. We evaluated transcriptome variation among testis, accessory gland, and the remaining whole-body preparations from males of the field cricket, *Teleogryllus oceanicus*. Non-normalized cDNA libraries from each tissue were sequenced on the Roche 454 platform, and a master assembly was constructed using testis, accessory gland, and whole-body preparations. A total of 940,200 reads were assembled into 41,962 contigs, to which 36,856 singletons (reads not assembled into a contig) were added to provide a total of 78,818 sequences used in annotation analysis. A total of 59,072 sequences (75%) were unique to one of the three tissues. Testis tissue had the greatest proportion of tissue-specific sequences (62.6%), followed by general body (56.43%) and accessory gland tissue (44.16%). We tested the hypothesis that tissues expressing gene products expected to evolve rapidly as a result of sexual selection—testis and accessory gland—would yield a smaller proportion of BLASTx matches to homologous genes in the model organism *Drosophila melanogaster* compared with whole-body tissue. Uniquely expressed sequences in both testis and accessory gland showed a significantly lower rate of matching to annotated *D. melanogaster* genes compared with those from general body tissue. These results correspond with empirical evidence that genes expressed in testis and accessory gland tissue are rapidly evolving targets of selection.

Field crickets (family Gryllidae) feature prominently in evolutionary, behavioral, and neurological studies, providing tractable and well-characterized nonmodel systems for research on sexual selection, speciation, and acoustic communication (Ewing 1989; Greenfield 2002; Huber *et al.* 1989). The field cricket *Teleogryllus oceanicus* has been used to study the genetic architecture of female preferences and male traits (Hoy *et al.* 1977), the effects of opposing natural and sexual selection on male song structure (Zuk *et al.* 1993), sperm competition (Simmons *et al.* 2007), the mechanics and neurophysiology of song production (Bennett-Clark 2003), insect learning and phenotypic plasticity (Bailey and Zuk 2009), and chemical signaling (Thomas and Simmons 2009), among others. Despite their widespread use in biological research, there are currently no comparative transcriptomic studies in gryllids.

The development of next-generation sequencing technology has created new opportunities to perform high-throughput analyses on organisms lacking a sequenced genome. In this study we characterized and compared the transcriptomes of three *T. oceanicus* tissues: testis, accessory gland, and general body. Genomic and/or transcriptomic data have been generated for only a handful of other gryllids, and to our knowledge these are restricted to expressed sequence tags from *Laupala kohalensis* neural tissue (Danley *et al.* 2007); *Allonemobius fasciatus*, *Gryllus firmus*, and *Gryllus pennsylvanicus* accessory gland tissue (Braswell *et al.* 2006); and *Gryllus bimaculatus* neural tissue (H. W. Horch, E. Sheldon, C. Cutting, and D. Riker, unpublished data).

A major goal of evolutionary studies is to integrate behavioral and physiological data with a better understanding of the sequence variation, regulation, and expression of genes underlying those processes. Such information provides an important step for resolving outstanding questions regarding the genomic and transcriptomic basis of complex traits (Boake *et al.* 2002). Our study therefore had two aims. The first was to develop transcriptome resources for *T. oceanicus*, and the second was to test whether patterns of annotation among the tissues reflect differences in the putative evolutionary histories of genes that are expressed in those tissues.

To address the first goal, we sequenced, assembled, and partially annotated a cross-tissue consensus transcriptome for *T. oceanicus*. A consensus transcriptome is a useful tool for single-nucleotide polymorphism (SNP) and microsatellite discovery, as well as expression profiling, and it provides a basis for comparative studies. We expected that tissues known to express genes that are under strong sexual selection, and therefore diverge rapidly, would show lower sequence similarity with genes sequenced in model organisms (Andrés *et al.* 2006, 2008; Attardo *et al.* 2010). Thus, to address the second goal, we compared annotation success in each tissue by using the well-studied and comparatively well-annotated model insect, *Drosophila melanogaster*. The rate of evolution of genes expressed in testis and accessory gland tissue should be greater compared with those expressed in body tissue because sexual selection is expected to act more strongly on, and cause rapid divergence in, gene products expressed in the context of mating, for example, proteins produced in the accessory glands (Walters and Harrison 2008, 2010; Wolfner 2002). In contrast, genes expressed in general body tissue are expected to show slower rates of divergence compared with homologous genes in other organisms. Rapidly evolving genes should be more difficult to annotate because of the lack of sequence similarity to other sequenced organisms, allowing us to test whether annotation success per tissue corresponds to expected differences in the strength of selection experienced by tissue-specific transcripts.

## Materials and Methods

### Samples and dissection

Adult *T. oceanicus*males and females were obtained from a large (>1000 individuals) outbred population derived originally from 50 females collected from a plantation in Carnarvon, Western Australia. No specific permits or permissions were required for the field collections. The population is seeded annually with >30 field caught females, and passes through four generations per year. Tissue was dissected from male accessory glands, testes, and the remaining muscle and tissues (the latter hereafter referred to as “whole body” tissue). Gut tissue was excluded from whole-body extractions to reduce contamination with foreign nucleic acids from ingested food or endosymbionts. Accessory gland tissue was extracted from 12 males, testis tissue from 20 males, and whole-body tissue from 6 males. The transcriptome of head tissue was sequenced as well, but we did not include it in our assembly or analyses because low RNA yield necessitated pooling the sexes. Tissues were immediately stored in RNAlater, with the exception of whole body tissue, which was immediately frozen at −80° in case RNAlater solution did not readily permeate the more bulky body samples. Total RNA was extracted using the PureLink RNA Mini kit (Invitrogen) according to the manufacturer’s instructions and stored at –80°.

Total RNA quality was checked spectophotometrically. To isolate mRNA before library preparation, each extract was precipitated with ethanol (Wallace 1987) and hybridized to oligo d(T) resins using a Dynabeads mRNA Purification kit (Invitrogen). This yielded a minimum of 2 μg of mRNA per tissue type, which was quality-checked as before and then used for 454 pyrosequencing.

### Sequencing

The GS FLX Titanium Sequencing Kit XLR70 (Roche) was used. Libraries were synthesized using random primer synthesis (EMD4Biosciences). The technique used *Eco*RI and *Hind*III linkers to create priming sites at the 3′ end of single-stranded mRNA transcripts. Double-stranded cDNA was synthesized using these primers, and subsequent digestion with *Eco*RI and *Hind*III produced fragments that were then ligated onto customized 454 adapters for the sequencing reaction. The resultant libraries were size-selected using Ampure XP beads (Agencourt) and Sizing Solution (Roche) according to the GS-FLX Titanim cDNA rapid library preparation standard method (Roche). cDNA libraries were not normalized before sequencing; thus, high abundance transcripts were more likely to be sequenced, and read numbers reflect transcript abundance. Each library was barcoded, pooled together in equal molar ratio, and sequenced as a single pool on a two-region gasket pico titer plate. Pyrosequencing was carried out following standard instrument operating procedures (Roche) at the Australian Genome Research Facility.

### Postsequencing processing

A ‘master’ assembly was constructed from reads pooled from accessory gland, general body and testis tissues, using Newbler’s runassembly program (v 2.6; Roche/454 Life Sciences) and the -cdna and -large options. Ribosomal RNA sequences were excluded at the assembly stage to avoid influences of tissue differences in RNA depletion in subsequent analyses. We submitted the master contigs to Deconseq (Schmieder and Edwards 2011) to check for and remove common contaminants. Very few sequences were identified as contaminants from *Homo sapiens*, *Mus musculus*, 18S, bacterial, archaeal, *Salmonella*, or viral genomes regardless of the thresholds set by the program. For example, there were only 68 sequences (0.16%) with 90% query coverage and 90% alignment identity. Sequences represented once among the reads of each tissue (singletons) were extracted using the sfffile and sffinfo commands of Newbler. The singletons were added to the tissue-specific contigs for the comparative annotation analysis. The count metrics per tissue were drawn as convex Venn diagrams using an online applet (Rodgers *et al.* 2010), and the generated figures were edited for clarity in PowerPoint.

The number of reads per contig was obtained for each tissue separately by mapping its reads of each tissue to the master assembly. The mapping enabled SNP detection for each tissue. We filtered Newbler’s ‘454AllDiffs.txt’ file to obtain ‘good’ SNPs for genotyping. Good SNPs are unlikely to be false positives, have a low chance of being close to another SNP, and are likely to occur at moderate frequencies in the population. The filtering criteria were implemented in PERL and were: a) 50 bp of uninterrupted flanking sequence, b) a minimum of 8 reads mapped to the contig containing the SNP and c) at least 15% minimum frequency for the minor allele.

We capitalised on the heterogeneity of annotation information available in online databases to test the prediction that BLASTx searching the *Drosophila* protein sequence database (FlyBase) would yield a greater proportion of annotated genes from body tissue because it expresses more conserved genes. We restricted this search to tissue-specific contigs, as identified from the mapping step, plus the singletons from each tissue. The BLAST analysis was performed in Blast2Go v.2.5.1 (Conesa *et al.* 2005). Chi-square analyses were used to test for significant differences among tissues in the proportion of tissue specific sequences yielding *Drosophila melanogaster* matches. We found an overall difference and performed pairwise post-hoc comparisons to separately test whether testis and accessory gland contigs were significantly less likely to produce matches than general body tissue. Bonferroni correction was applied to significance values to account for multiple comparisons. Chi-square analyses were performed using Minitab v.12.21.

## Results

### Sequencing and assembly

**Figure 1:**
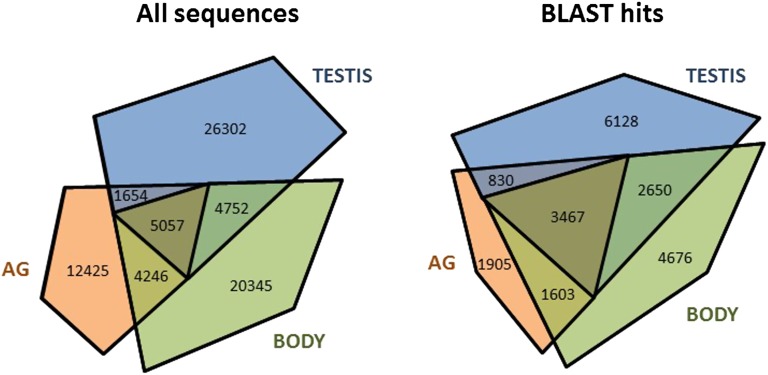
Distribution of sequences among tissues. The distribution of all sequences (contigs and singletons) mapping to each tissue is shown on the left, and the distribution of sequences from each tissue with successful BLASTx hits is shown on the right.

We used Roche 454 pyrosequencing to generate sequences from general body, testis, accessory gland, and head (neural) tissues. Extractions yielded between 110 and 155 μg of total RNA from the four tissue types, and sequencing produced approximately 1.2 million reads in total (940,200 when head tissue is excluded; Table 1). All reads have been deposited in the National Center for Biotechnology Information Sequence Read Archive under project accession number SRP007757. Roughly 485,000 reads were then assembled into approximately 42,000 contigs, of which 12,332 were greater than 500 bp in length (Table 2, Figure 1). The remaining reads did not assemble, most likely due to poor read quality. The proportion of reads from each tissue that was used in the master assembly varied among tissues from 42.88 to 84.66% (Table 1). This variation may reflect differences in quality of mRNA extractions as equimolar ratios of cDNAs were sequenced on the Illumina plate.

**Table 1:** Summary metrics for the assembly and mapping of reads from general body, testis, and accessory gland tissues to the master assembly

		Accessory Gland	Testis	General Body	Sum
Reads	Total*^a^*	288,814	293,976	357,410	940,200
	After trimming	215,174	260,647	258,256	734,077
Assembly	Assembled*^b^*	92,263	220,661	174,789	487,713
		42.88%	84.66%	67.68%	
Mapping	Fully mapped	32.78%	50.59%	41.14%	
	Partially mapped*^c^*	11.97%	12.82%	12.8%	
	Repeat reads mapped*^d^*	18.12%	3.61%	5.7%	
	Chimeric reads*^e^* mapped	30.41%	27.52%	28.86%	
	% trimmed reads mapped	93.27%	94.54%	93.5%	

a Reads (.sff files) have been deposited in the NCBI Sequence Read Archive.

b The number and proportion of reads in each tissue that contributed to the master assembly.

c Partially mapped reads do not fully map to a contig.

d Repeat reads map to multiple contigs.

e Chimeric reads have different parts mapping to separate contigs.

**Table 2:** Contig metrics from the *de novo* assembly based on transcriptome data pooled from three different *Teleogryllus oceanicus* tissues

Metric	Number (>500 bp)	Total Number
Contig number	12,332	41,965
Average contig size	894	459
Median contig size	727	373
Median read depth	73	2.4
Mean read depth	176	9
Largest contig	10,718	

Read depth was skewed toward very small numbers, with only 1424 sequences mapped by >30 reads.

Tables 1 and 2 show summary statistics on the master transcriptome assembly and the sequence quality, read numbers, and contig information for each tissue. The master assembly can be accessed in Supporting Information, File S2. This file contains each contig and the number of reads in each tissue, plus gene homology information where available. RNA yield varied between tissues, and it appears to be associated with variation in the sequencing and assembly metrics among tissues. For example, testis tissue showed the greatest mRNA extraction yield of the four tissues (>5 µg of mRNA), and it contributed the greatest proportion of reads to the construction of contigs in the master assembly (ca. 85%). Accordingly, testis tissue had the greatest proportion (ca. 51%) of reads fully mapping to the master assembly (Table 1).

There were 17,450 high confidence differences (SNPs and indels, most were SNPs) resulting from mapping the reads of each tissue to the master assembly. The master contigs contain about 19 million bases, resulting in a SNP density ranging from roughly 9.1 × 10^−4^ to 21.8 × 10^−4^ SNPs per bp, depending on SNP confidence. Approximately 3160 high-confidence, potentially scorable SNPs were identified within 1633 contigs. The SNP variants, and their positions on the master contigs, are provided in File S1A−C. The tissue(s) which map to those contigs are shown in File S1D, and the distribution of the SNPs among the tissues is shown in File S1E (the majority of SNPs were tissue-specific).

### Comparisons with other species

**Figure 2:**
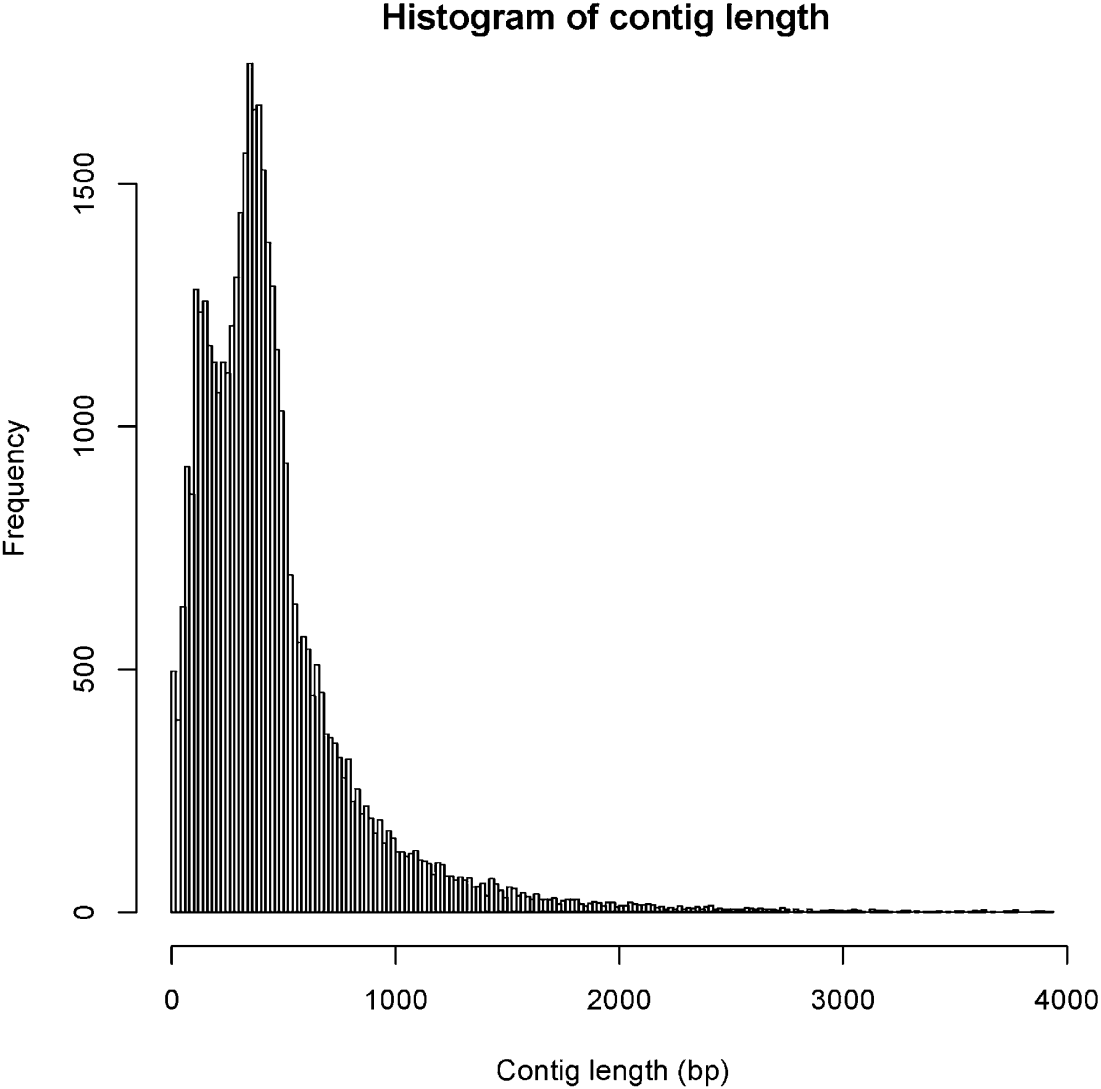
Histogram of contig lengths. Only contigs <4000 bp are shown. The data have a long tail with no peaks reaching 10,718 bp.

**Figure 3:**
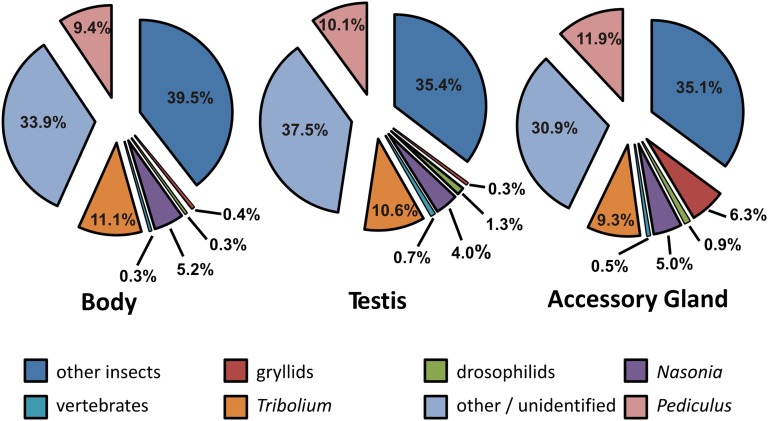
Species matches to tissue-specific sequences (contigs and singletons). The percentages indicate the top BLASTx matches for each species category.

**Figure 4:**
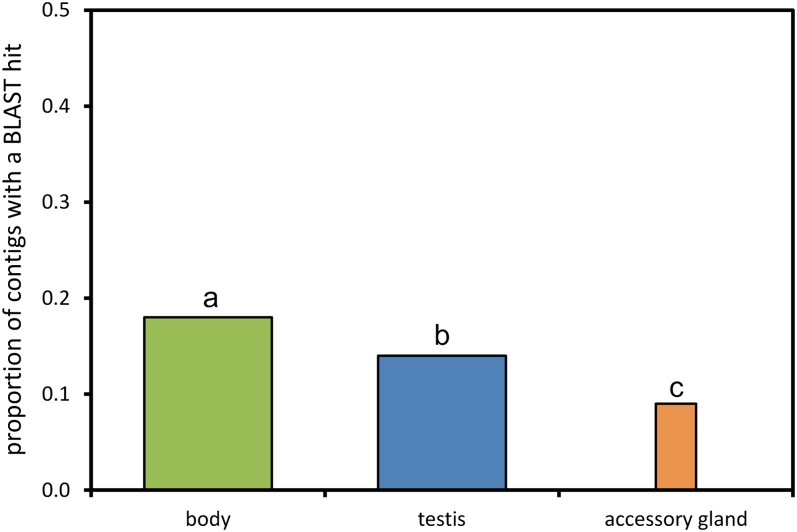
The proportion of tissue-specific sequences (contigs and singletons) with a BLASTx match to *Drosophila melanogaster* proteins in FlyBase. Column widths are proportional to the total number of sequences. Tissues that were significantly different do not share a small letter: both testis and accessory gland sequences showed a lower proportion of matches to *D. melanogaster* genes compared with general body tissue and also were significantly different from one another (see *Results* section for details).

Our second goal was to use publicly available databases to compare annotation success among tissue-specific sequences (contigs and singletons), with the expectation that annotation of more rapidly evolving genes will be less likely due to a lack of sequence similarity with related organisms. The distribution of species matches associated with the top BLASTx hits was not the same among the tissues (Figures 2 and 3). It was roughly similar for body and testis sequences, whereas accessory gland sequences showed more matches to other insects, and a considerable portion of the top hits (more than 6%) matched sequences from other gryllid species (Figure 3). These differences likely reflect a bias in the research foci of labs that use gryllids in gene expression studies (Andrés *et al.* 2008). To assess whether the sequences that were only detected in testis and accessory gland tissues diverged so rapidly that BLASTx fails to detect homology with the model insect *Drosophila melanogaster*, we quantified, for each tissue, the proportion of sequences with BLASTx hits that matched annotated proteins in the *D. melanogaster* protein sequence database curated in FlyBase. *D. melanogaster* is the most closely related insect to *T. oceanicus*, with the best-annotated genome. Narrowing our search to a standardized and genome-sequenced model insect species reduced the chances that uneven annotation among tissues would influence the results, because annotation comparisons that included nonmodel organisms could be affected by the focus of some labs on particular tissues in particular species. Overall, the three tissues differed significantly in the proportion of tissue-specific sequences matching to D. melanogaster proteins (Figure 4; χ^2^ test: χ^2^ = 485.31, df = 2, *P* < 0.001). In pairwise comparisons using general body tissue as a baseline, testis tissue showed a significantly lower proportion of matches to *D. melanogaster* genes (χ^2^ test: χ^2^ = 143.12, df = 1, *P* < 0.001). Similarly, accessory gland tissue showed a significantly lower proportion of matches to *D. melanogaster* genes (χ^2^ test: χ^2^ = 469.58, df = 1, *P* < 0.001). Finally, accessory gland had a lower rate of matching than testis tissue (χ^2^ test: χ^2^ = 16.82, df = 1, *P* < 0.001). All comparisons remained significant after Bonferroni correction for multiple testing (α = 0.013).

## Discussion

We have developed a necessary resource for future gryllid studies. Roche 454 pyrosequencing can provide thorough representation of mRNA transcripts present in a cell or tissue (Morozova and Marra 2008; Wall *et al.* 2009; Wheat 2010) despite producing fewer reads per run, and our results confirm the utility of the platform for identifying and characterizing transcriptomic variation among tissue types in a frequently studied insect, the field cricket *T. oceanicus*. The availability of sequences from multiple tissues, and potentially scorable SNPs and microsatellites, can form the basis for future studies of different individuals or different species, as has been done in ecological genomic studies of birds (Künstner *et al.* 2010), cichlid fish (Elmer *et al.* 2010), and butterflies (Vera *et al.* 2007). Perhaps more significantly, the assembly of a reference transcriptome for *T. oceanicus* addresses one of the major challenges to comparative transcriptomics in ecological, behavioral, or evolutionary studies of nonmodel organisms: a lack of transcriptome sequence data. There was a large degree of tissue specificity in sequences (>50%). Taken together, our results demonstrate considerable transcriptomic differences among distinct *T. oceanicus* tissue types. Althouggh such specificity was expected for testis and accessory gland tissue given their specialized functions, we found a large proportion of tissue-specific transcripts in general body tissue as well. Testis tissue showed both the largest number and greatest proportion of tissue-specific sequences.

The proportion of accessory gland transcripts that was possible to annotate was lower than body and testis transcripts, a pattern consistent with the rapid evolution of reproductive proteins and the high rates of both functional and genetic divergence in accessory gland proteins that have been documented in crickets and other insects (Andrés *et al.* 2006, 2008; Attardo *et al.* 2010; Boake *et al.* 2002; Walters and Harrison 2008, 2010; Wolfner 2002). Transcripts from testis tissue also showed lower sequence similarity to annotated *D. melanogaster* genes than body tissue. Rapid evolution by sexual selection may partially explain annotation success differences. As transcriptomic information on seminal protein gene expression accumulates in *T. oceanicus* and other gryllid species, future studies would benefit from comparative approaches that incorporate tissue-specific expression profiling.

The different annotation success among the tissues supports the prediction that tissues known to express rapidly evolving genes will yield tissue-specific contigs that are more difficult to match to annotated genes in closely related organisms. This observation underscores a key difficulty in performing expression profiling studies targeting traits or proteins that are expected to experience rapid evolution due to sexual selection or other pressures, namely that drawing useful comparisons among species or even within species is hampered by the fact that annotation information will be more severely limited for rapidly evolving genes. However, as we have demonstrated, it may be possible to capitalize on this limitation by extrapolating information about the rate of evolution of genes on a transcriptome-wide basis by quantifying the availability of annotation data for transcripts recovered from different tissues, individuals or species.

## Supplementary Material

Supporting Information
