# Supplementary material for: Tissue-Specific Transcriptomics in the Field Cricket Teleogryllus oceanicus
Source: G3 (Bethesda). 2013 Feb 1;3(2):225–30. doi: 10.1534/g3.112.004341 (PMC3564983; doi:10.1534/g3.112.004341)
Supplement: Supporting Information [file supp_3_2_225__index.html]

Supporting Information 

# Tissue-Specific Transcriptomics in the Field Cricket *Teleogryllus oceanicus*

## Supporting Information for Bailey *et al.*, 2013

**Files in this Data Supplement:**

- Supporting Information - Files S1 and S2 (PDF, 92 KB)
- File S1 - Folder containing: Files S1a-c) SNPs identified by mapping the general body, testis and accessory gland reads; File S1d) list of the tissues whose reads contributed to the identification of the above SNPs; File S1e) A Venn diagram of the distribution of SNPs among the tissues they were identified from (.zip, 206 KB)
- File S2 - The full sequence of all 41,962 contigs of the master assembly, the name of the first BLASTx hit and the number of reads mapped from each tissue (.zip, 6.4 MB)
