# Supplementary material for: Tissue-Specific Transcriptomics in the Field Cricket Teleogryllus oceanicus
Source: G3 (Bethesda). 2013 Feb 1;3(2):225–30. doi: 10.1534/g3.112.004341 (PMC3564983; doi:10.1534/g3.112.004341)
Supplement: Supporting Information [file supp_3.2.225_004341SI.pdf]

G3· Genes | Genomes | Genetics

Tissue-Specific Transcriptomics in the Field Cricket Teleogryllus oceanicus

Nathan W. Bailey*[,1], Paris Veltsos*[,1], Yew-Foon Tan[§,¥], A. Harvey Millar[¥], Michael G. Ritchie*, Leigh W. Simmons[§]

* Centre for Biological Diversity, School of Biology, University of St Andrews, St Andrews, Fife KY16 9TH, United Kingdom

[§] Centre for Evolutionary Biology, School of Animal Biology (M092), The University of Western Australia, Crawley 6009, Australia

[¥] Centre for Comparative Analysis of Biomolecular Networks, The University of Western Australia, Crawley 6009, Australia

Data deposited in: National Center for Biotechnology Information Sequence Read Archive under project accession number SRP007757

File S1

Available for download at http://www.g3journal.org/lookup/suppl/doi:10.1534/g3.112.004341/-/DC1

The folder contains:

Files S1a, 1b, 1c: agSNPs.txt, bodySNPs.txt, testisSNPs.txt

These files contain SNPs identified by mapping the general body, testis and accessory gland reads. They have been filtered to high-confidence SNPs (see main text). The headers and their explanations are:

Table S1 SNP file header

Reference	Start	End	Ref	Var	Total	Var
The contig	Start position	End position	SNP variant 1	SNP variant 2	Total SNPs	% SNP variant 2

File S1d: SNPsVennData.txt

This file indicates the tissues whose reads contributed to the identification of the above SNPs.

File S1e: SNPvenn.pdf

A Venn diagram of the distribution of SNPs among the tissues they were identified from.

File S2

Available for download at http://www.g3journal.org/lookup/suppl/doi:10.1534/g3.112.004341/-/DC1.

This folder contains the 3TissueContigsInfo.txt file, which contains the full sequence of all 41,962 contigs of the master assembly, the name of the first BLASTx hit and the number of reads mapped from each tissue. These sequences should be useful as a scaffold for assembly when additional next-generation sequencing data are available.
