# Supplementary material for: Tissue-Specific Transcriptomics in the Field Cricket Teleogryllus oceanicus
Source: G3 (Bethesda). 2013 Feb 1;3(2):225–30. doi: 10.1534/g3.112.004341 (PMC3564983; doi:10.1534/g3.112.004341)
Supplement: Supporting Information [file supp_3.2.225_FileS1.zip › FileS1/FileS1.pdf]

File S1

Available for download at http://www.g3journal.org/lookup/suppl/doi:10.1534/g3.112.004341/-/DC1

The folder contains:

Files S1a, 1b, 1c: agSNPs.txt, bodySNPs.txt, testisSNPs.txt

These files contain SNPs identified by mapping the general body, testis and accessory gland reads. They have been filtered to high-confidence SNPs (see main text). The headers and their explanations are:

Table S1 SNP file header

Reference	Start	End	Ref	Var	Total	Var
The contig	Start position	End position	SNP variant 1	SNP variant 2	Total SNPs	% SNP variant 2

File S1d: SNPsVennData.txt

This file indicates the tissues whose reads contributed to the identification of the above SNPs.

File S1e: SNPvenn.pdf

A Venn diagram of the distribution of SNPs among the tissues they were identified from.
