# Supplementary figures and images for: Tissue-Specific Transcriptomics in the Field Cricket Teleogryllus oceanicus
Source: G3 (Bethesda). 2013 Feb 1;3(2):225–30. doi: 10.1534/g3.112.004341 (PMC3564983; doi:10.1534/g3.112.004341)

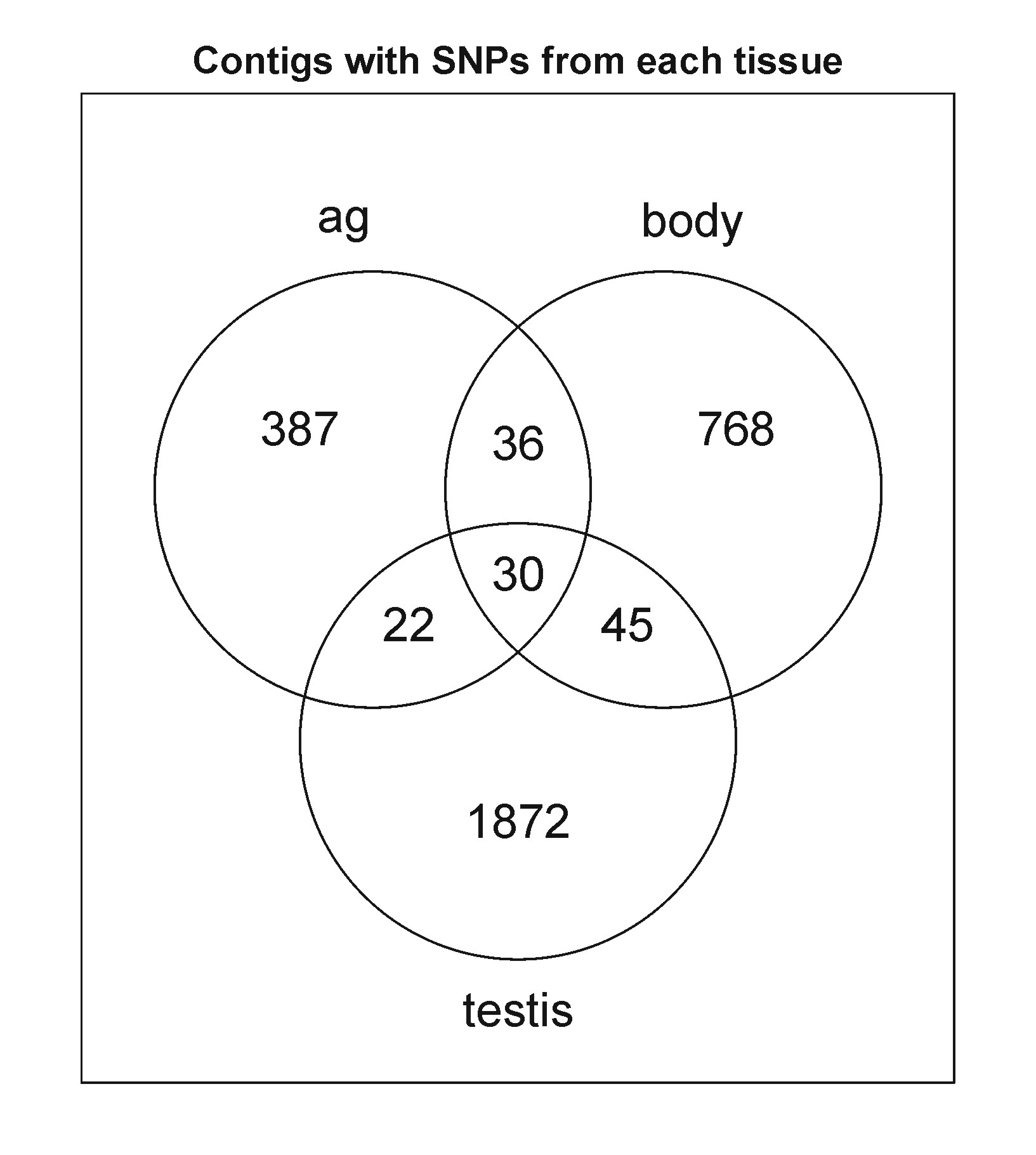

Supplement: Supporting Information [file supp_3.2.225_FileS1.zip › FileS1/Supplemental file 1e.jpg]
