# Supplementary material for: Tissue-Specific Transcriptomics in the Field Cricket Teleogryllus oceanicus
Source: G3 (Bethesda). 2013 Feb 1;3(2):225–30. doi: 10.1534/g3.112.004341 (PMC3564983; doi:10.1534/g3.112.004341)
Supplement: Supporting Information [file supp_3.2.225_FileS2.zip › FileS2/FileS2.pdf]

File S2

Available for download at http://www.g3journal.org/lookup/suppl/doi:10.1534/g3.112.004341/-/DC1.

This folder contains the 3TissueContigsInfo.txt file, which contains the full sequence of all 41,962 contigs of the master assembly, the name of the first BLASTx hit and the number of reads mapped from each tissue. These sequences should be useful as a scaffold for assembly when additional next-generation sequencing data are available.
